# PROTOCOL: Assessment of outcome reporting bias in studies included in Campbell systematic reviews

**DOI:** 10.1002/cl2.1332

**Published:** 2023-05-25

**Authors:** Julia H. Littell, Dennis M. Gorman, Jeffrey C. Valentine, Therese D. Pigott

**Affiliations:** ^1^ Graduate School of Social Work and Social Research Bryn Mawr College Bryn Mawr Pennsylvania USA; ^2^ Department of Epidemiology & Biostatistics and School of Public Health Texas A&M University College Station Texas USA; ^3^ Department Counseling and Human Development University of Louisville Louisville Kentucky USA; ^4^ School of Public Health Georgia State University Atlanta Georgia USA

## Abstract

This is the protocol for a Campbell systematic review. The objectives are as follows: To identify methods used to assess the risk of outcome reporting bias (ORB) in studies included in recent Campbell systematic reviews of intervention effects. The review will answer the following questions: What proportion of recent Campbell reviews included assessment of ORB? How did recent reviews define levels of risk of ORB (what categories, labels, and definitions did they use)? To what extent and how did these reviews use study protocols as sources of data on ORB? To what extent and how did reviews document reasons for judgments about risk of ORB? To what extent and how did reviews assess the inter‐rater reliability of ORB ratings? To what extent and how were issues of ORB considered in the review's abstract, plain language summary, and conclusions?

## 1 BACKGROUND

### 1.1 The problem

Systematic reviewers are expected to assess methodological characteristics and risks of bias within the studies included in a review to gauge the credibility and certainty of the evidence these studies provide (Campbell Methods Group, 2019a, 2019b; Higgins et al., 2019; Page et al., 2019; Page et al., 2021a, 2021b; Sterne et al., 2019). Here, we are concerned with how reviewers assess the risk of selective reporting of outcomes in studies included within systematic reviews (SRs) of intervention effects. We begin with a brief overview of (a) what is known about outcome reporting bias (ORB) in primary studies, (b) the threat this bias poses to the validity of intervention reviews, (c) steps to improve transparency of reporting and facilitate detection of reporting biases in primary studies, and (d) methods reviewers use to detect and assess ORB in studies in their reviews.

ORB is the selective reporting or nonreporting of research results, based on their direction and/or statistical significance. In contrast to publication bias, which refers to the selective publication or nonpublication of entire studies, ORB involves selective reporting of outcomes within studies. Most evaluation studies include multiple outcomes and endpoints; hence, they have multiple results. Some results may be fully reported (with data sufficient to support meta‐analysis [MA]), while other results may be under‐reported (with missing information), and some results may not be mentioned at all.

Reporting patterns are not random. There is a large body of empirical literature that shows that positive results (those that confirm prior expectations) and statistically significant results are more likely to be fully reported—in both unpublished and published papers—compared with equally valid negative and null results (Dwan et al., 2008; Dwan et al., 2013; Norris et al., 2012; Pigott et al., 2013; Song et al., 2009, Song et al., 2010). For example, in a study of dissertations on educational interventions, Pigott and colleagues (Pigott et al., 2013) compared 79 publications to the dissertations upon which they were based; only 24% (19 publications) included all the outcomes described in the dissertation, and the odds of publication were 2.4 times greater for statistically significant versus non‐significant outcomes.

Chalmers (1990) argued that under‐reporting of research results is form of scientific misconduct, yet many investigators “seemed generally unaware of the implications for the evidence base of not reporting all outcomes” (Smyth et al., 2011, p. 1). Trained to view statistical significance as an indicator of important or noteworthy results, some researchers may not understand that selective reporting introduces bias into the literature and impedes access to important unreported and under‐reported empirical results. Indeed, Song and colleagues (2009) found that investigators are the main source of reporting bias, because this bias tends to arise early in the dissemination process, before results are submitted for publication. Peer reviewers and journal editors are other potential sources of influence on reporting (Mahoney, 1977), but these influences are poorly understood (Tennant & Ross‐Hellauer, 2020). Song and colleagues (2009, 2010) found little evidence that selection bias occurred after manuscripts were submitted to journals, although Goldacre and colleagues (2019) found that some journal editors do not understand ORB well and some are reluctant to correct misreporting.

ORB poses a potential threat to the validity of SRs and MA, because it introduces bias in terms of the range of results that are available from included studies. When results are fully reported (e.g., with valid Ns, means and standard deviations or proportions for all subgroups), they can be included in MA. Partial reporting of results (e.g., simply stating that a finding was not statistically significant and providing little or no additional information beyond this) and non‐reporting of results (failure to mention non‐significant or negative results at all) makes their inclusion in MA impossible without additional information from the authors or assumptions by the meta‐analysts. At best, this represents a loss of information that could contribute to more powerful tests of hypotheses, including moderator analyses, for which statistical power tends to low (Valentine et al., 2010). At worst, the results of the SR and MA will be biased, usually by inflating estimates of beneficial effects and underestimating potential harms.

There is evidence that reporting biases affect results of SRs (Song et al., 2010). Kirkham and colleagues (Kirkham et al., 2010) found that more than half (157) of 283 Cochrane reviews did not include all outcomes of interest from all eligible trials, and one‐quarter (70 reviews) were missing at least 50% of the relevant data. Sensitivity analysis showed that the treatment effect was overestimated by 20% or more in almost one‐fourth (19) of the 81 reviews that had only one MA. Almost one‐fifth (8) of 42 MAs with a statistically significant effect became non‐significant after adjustment for ORB and more than one‐fourth (11 MAs) overestimated the treatment effect by 20% or more. ORB appears may be even more pronounced in reviews of adverse effects; for example, most (79 of 92) Cochrane reviews did not include all the data on the main harm outcome of interest (Saini et al., 2014).

To improve transparency and facilitate later detection of publication and reporting biases, trialists have been encouraged to deposit detailed protocols, describing all planned outcome measures and endpoints, into a public registry before enrollment into the study begins. Some funders and journals require prospective public registration of studies as a condition of funding or publication (De Angelis et al., 2004), but enforcement is weak and prospective registration is uneven (Alayche et al., 2022; Chan et al., 2017; Lamberlink et al., 2022). For example, Norris and colleagues (2012) examined three samples of randomized controlled trials (RCTs) (from three comparative effectiveness reviews) and found that 29% to 76% of the RCTs were registered; 24% to 42% were registered after the trial had been completed; outcomes were changed in the registration records of 17% to 42% of the trials; and publications did not consistently mention trial registration when it existed. Bradley et al. (2016) examined 112 psychotherapy trials published between 2010 and 2014; almost 60% (67) were registered, but only one‐quarter (27) were prospectively registered, only 13 (12%) were correctly registered and reported, and only five were free of ORB. In a study of 136 registered trials in behavioral health, Taylor and Gorman (2022) found that only half were prospectively registered and only 16 published manuscripts reported outcomes and methods consistent with their registrations. These studies show that public registration has not been sufficient to ensure adequate documentation and full reporting of trials.

### 1.2 Methods for detecting ORB

In contrast to methods for assessing and adjusting for publication bias (e.g., Rothstein et al., 2005; Schwarzer et al., 2010; Song et al., 2010), methods for detecting and adjusting for ORB are not well developed. Page and colleagues (2018) identified 18 tools that included assessment of risk of reporting bias and identified limitations of these tools in terms of their scope, guidance, and measurement properties.

ORB can, in some cases, be impossible for individuals outside of the research team to detect. It is almost always difficult to detect. Of course, it would be easier to detect ORB if every study had a list of pre‐registered outcomes and analyses. Reviewers could then compare published reports with the pre‐registered list. Any unreported pre‐registered outcomes, or newly reported unregistered outcomes, would then be viewed with heightened suspicion and, research teams could be asked directly about these discrepancies. But in practice, protocols are not well‐established in most disciplines, are not written at an operational level (i.e., specific measurement instruments, timings, sources of data, and analyses are not all pre‐specified), and protocols are sometimes registered retrospectively or altered after initial plans have changed. Therefore, investigating the plausibility of ORB in most cases requires relying on clues left in the paper trail, triangulating across multiple reports of the same study, and employing professional judgment regarding the likelihood that a particular outcome was indeed measured. These are high‐inference tasks that will usually not support confident judgments about the presence or absence of ORB.

Below we describe three prominent approaches: (1) assessment of ORB within broader risk of bias (ROB) assessments, (2) the Outcome Reporting Bias In Trials (ORBIT) approach, and (3) the Campbell Collaboration's Methodological Expectations for Campbell Collaboration Intervention Reviews (MECCIR).

#### 1.2.1 ROB tools

When ORB is assessed in SRs, it is usually one component in a larger set of structured assessments of several distinct types of bias (e.g., selection bias, detection bias, performance bias, attrition bias). In the past, this type of work was often termed study quality assessment (or methodological quality assessment). Risk of bias rubrics now focus more clearly on issues that may affect the credibility of conclusions that can be drawn from individual studies and SRs. This structured critical appraisal of included studies is an important feature of SRs.

Because most ROB tools require high‐inference judgments, they are often conducted by two independent raters, who then compare notes and resolve any discrepancies.

Shown in Table 1, several risk of bias (ROB) tools have been developed for use in SRs. Cochrane's initial tool (ROB 1; Higgins & Green, 2011) and its successor (ROB 2; Sterne et al., 2019) were designed to assess risks of bias in RCTs. ROBINS‐I (Sterne et al., 2016) assesses ROB in non‐RCTs as well as RCTs, if both types of studies are included within a review. (All three tools are available at https://riskofbias.info). Some reviewers found these tools burdensome and they have poor to moderate interrater reliability (Hartling et al., 2009; Minozzi et al., 2020; Jeyaraman et al., 2020).

**Table 1:** Examples of ROB tools that include assessment of ORB.

Tool	Types of studies	# of ROB domains assessed	Assessment method for ORB	Ratings	Inter‐rater reliability (***κ***)
Cochrane ROB 1 (Higgins & Green, 2011)	RCTs	6	“State how selective outcome reporting was examined and what was found.”	Low risk, unclear, or high risks of bias	0.27 overall, 0.13 ORB (Hartling et al., 2009; *k* = 163)
Cochrane ROB 2 (Sterne et al., 2019)	RCTs and quasi‐RCTs	5	3 signaling questions and an algorithm	Low risk, some concerns, High risk	0.15 overall, 0.30 ORB (Minozzi et al., 2020; *k* = 70)
ROBINS‐I (Sterne et al., 2016)	RCTs and non‐RCTs	7	3 signaling questions	Low risk, moderate risk, serious risk, critical risk, no info	0.00 overall, 0.54 ORB (Jeyaraman et al., 2020; *k* = 44)
EPOC (2017)	Studies with control groups	9	“Score ‘Low risk’ if there is no evidence that outcomes were selectively reported (e.g., all relevant outcomes in the methods section are reported in the results section). Score ‘High risk’ if some important outcomes are subsequently omitted from the results. Score ‘Unclear risk’ if not specified in the paper.”		Unknown
EPOC (2017)	ITS	7			Unknown

Abbreviations: ORB, outcome reporting bias; RCT, randomized controlled trial.

Cochrane's EPOC group (EPOC, 2017) developed separate ROB tools for two groups of studies: (1) those that use control groups and (2) interrupted time series (ITS) designs. Scoring instructions for ORB are identical in these two tools. We found no information on their reliability.

#### 1.2.2 ORBIT

Outcome Reporting Bias In Trials (ORBIT) is the most elaborate system for assessing ORB (see https://outcome-reporting-bias.org). Dwan, Kirkham, and colleagues (Dwan et al., 2010; Kirkham et al., 2018) published tutorials for using ORBIT tools. Separate ORBIT classifications systems were developed for benefits and adverse effects. The former is shown in Table 2. It assesses “risk of bias arising from the lack of inclusion of non‐significant results…” For adverse outcomes, high risk of bias occurs when “data were presented or suppressed in a way that would mask the harm profile of particular interventions” (https://outcome-reporting-bias.org/HarmOutcomes).

**Table 2:** ORBIT classifications (benefit outcomes).

Classification	Description	Level of reporting	Risk of bias^a^
*Clear that outcome was measured and analyzed*			
A	Trial report states that outcome was analyzed but only reports that result was not significant (typically stating *p*‐value > 0.05).	Partial	High risk
B	Trial report states that outcome was analyzed but only reports that result was significant (typically stating *p*‐value < 0.05).	Partial	No risk
C	Trial report states that outcome was analyzed but insufficient data were presented for the trial to be included in meta‐analysis or to be considered to be fully tabulated.	Partial	Low risk
D	Trial report states that outcome was analyzed but no results reported.	None	High risk
*Clear that the outcome was measured*			
E	Clear that the outcome was measured. Judgment says outcome likely to have been analyzed but not reported because of non‐significant results.	None	High risk
F	Clear that the outcome was measured. Judgment says outcome unlikely to have been analyzed.	None	Low risk
*Unclear whether the outcome was measured*			
G	Not mentioned but clinical judgment says likely to have been measured and analyzed but not reported on the basis of non‐significant results.	None	High risk
H	Not mentioned but clinical judgment says unlikely to have been measured at all.	None	Low risk
*Clear that the outcome was not measured*			
I	Clear that the outcome was not measured	NA	No risk

Abbreviation: ORBIT, Outcome Reporting Bias In Trials.

^a^ Risk of bias arising from the lack of inclusion of non‐significant results when a trial was excluded from meta‐analysis or not fully reported in a review because the data were unavailable.

*Source*: https://outcome-reporting-bias.org/BenefitOutcomes

Some users have reported difficulties using the ORBIT approach. Norris and colleagues (Norris et al., 2012) noted that ORBIT (1) does not classify outcomes that were pre‐specified and fully reported (these should be classified as low risk or no risk) and (2) does not cover some types of ORB, such as (a) reporting of outcomes that were not prespecified and (b) changes in data measurement or analysis plans (including “data dredging”).

The ORBIT tool requires high‐inference judgments (including clinical judgments). It appears to emphasize discrepancies within study reports, rather than comparisons between study protocols and reports. We found no information on inter‐rater reliability of ORBIT ratings.

#### 1.2.3 MECCIR

The Campbell Collaboration's MECCIR standards indicate that assessment and documentation of study‐level ROB is mandatory in Campbell reviews on interventions, but the components of these ROB assessments are not specified (Campbell Methods Group, 2019a, 2019b). Campbell reviews may use ROB tools, ORBIT, or other approaches for assessment of ORB. A study of 96 Campbell reviews published from 2011 through 2018 showed that:

- 82% (79) of the reviews described the tool used for ROB assessments,
- 63% (60) described the methods used to assess ROB,
- 79% (76) reported results of ROB assessments, and
- 73% (70) took ROB into account in interpretation of results (Wang et al., 2021).

To our knowledge, there are no studies of methods used to assess ORB in Campbell reviews.

### 1.3 How these methods might work

Ideally, systematic reviewers would obtain a prospectively registered protocol (from a public trial registry or from study authors) for each included study, and then compare the pre‐specified measures and endpoints described in the protocol with the results and endpoints reported in subsequent papers to detect ORB. If all prespecified measures are fully reported at all endpoints, then there is no ORB. The potential for ORB arises when reports include a subset of the pre‐specified outcomes, some endpoints are not reported, unspecified outcomes are added, or changes are made in measurement or analysis plans.

As the number of differences between pre‐planned and reported results increases, the potential risk of ORB increases. However, more convincing evidence of ORB is sometimes found in (a) researchers’ explicit statements that their reports focus on statistically significant and/or positive results, or in (b) patterns of reporting that differ for significant versus non‐significant (or positive vs. other) results. For example, some reports provide full statistical details for significant results, but only mentioned non‐significant results in the text or partially report these results in tables.

Many studies do not have prospective protocols, and in these cases, reviewers must rely on retrospective protocols or descriptions provided in the methods sections of study reports. There are gaps and ambiguities in guidance on the use of study protocols and trial registries in SRs (Boden et al., 2017). And there is little information on how reviewers handle different sources of evidence of ORB.

### 1.4 Why it is important to do this review

ORB is a pernicious problem in the scientific literature. It appears to affect results of SRs, and it may be one of the most difficult types of bias to assess in SRs. Tools to help reviewers assess ORB are under‐developed or unwieldy. Knowledge about whether and how reviewers attempt to assess ORB could lead to recommendations for improving ORB assessments in future SRs.

There are no SRs of methods used to assess ORB in Campbell reviews. Wang and colleagues (Wang et al., 2021) considered whether and how Campbell reviews assessed risks of bias but did not examine assessment of risks of specific types of bias, such as ORB.)

ORB ratings appear to be perfunctory in some reviews, indicating that there is room for improvement in methods of assessing ORB. For example, a prominent Campbell review (Gaffney et al., 2021) assessed ORB as “low risk” in most included studies but provided no support for these ratings; independent attempts to replicate these ratings (using the review's stated criteria) with 41 studies showed very low (7%) agreement (*κ* = 0.003; Littell & Gorman, 2022).

ORB also arises within SRs, when reviewers report their result selectively. For example, Shah and colleagues (Shah et al., 2020) documented discrepancies in outcome reporting (DOR) in a sample of 350 Cochrane reviews (published between 2007 and 2014), by comparing the protocols for these SRs with completed reports. Forty‐three percent (150) of 350 review and protocol pairs included DOR, and 23% had a high risk of ORB, as changes in reported outcomes were made after results were known. Our review will focus on whether and how reviewers assessed ORB in the studies they included, and not on whether the results of reviews were selectively reported.

## 2 OBJECTIVES

To identify methods used in recent Campbell SRs of intervention effects to assess the risk of ORB in included studies, we will attempt to answer the following questions:

- What proportion of recent Campbell reviews included assessment of ORB?
- How did recent reviews define levels of risk of ORB? (What categories, labels, and definitions did they use?)
- To what extent and how did these reviews use study protocols as sources of data on ORB?
- To what extent and how did reviews document reasons for judgments about risk of ORB?
- To what extent and how did reviews assess the inter‐rater reliability of ORB ratings?
- To what extent and how were issues of ORB considered in the review's abstract, plain language summary, and conclusions? (MECCIR C73, R96, R100).

## 3 METHODS

### 3.1 Criteria for considering studies for this review

We will include all SRs (including newly update reviews) that

- included primary studies of intervention effects and
- were published in *Campbell Systematic Reviews* between 1 January 2020 and 31 December 2022.

We limit the focus to reviews of primary studies of intervention effects, because (a) most of the tools for assessing ORB were developed for these kinds of reviews and (b) Campbell has guidelines for the conduct and reporting of these types of reviews, but not for other kinds of reviews (Campbell Methods Group, 2019a; Campbell Methods Group, 2019b). We will exclude overviews of reviews, in which the primary unit of analysis is the review.

We will only include SRs published after the release of new guidance for the assessment of ORB (Sterne, Savović, Page, et al., 2019; Campbell Methods Group, 2019a, 2019b). This excludes SRs published before 2000.

### 3.2 Search methods for identification of studies

We will search the Campbell Library website, using the structured online form developed for this purpose (https://www.campbellcollaboration.org/component/jak2filter/?Itemid=1352&issearch=1&isc=1&category_id=101&xf_8[0]=3&ordering=publishUp).

We will use filters for

- publication dates (from January 1, 2020 through December 31, 2022) and
- type of document (Reviews only; see (Supporting Information: Appendix 1).

A preliminary search produced 32 records of Campbell reviews published in 2020 and 2021.

### 3.3 Data collection and analysis

#### 3.3.1 Selection of reviews

Working independently, two screeners will scan each review to determine whether it meets our eligibility criteria, using the screening tool provided in Supporting Information: Appendix 2. Screeners will compare notes and resolve any discrepancies, involving a third member of the review team, if necessary. Selection decisions will be documented, with specific reasons for exclusion for each excluded review. Results of the selection process will be reported in a PRIMSA flow diagram and in tables of include and excluded reviews.

#### 3.3.2 Data extraction and management

For each included review, data extraction will be only conducted by team members who were not co‐authors of the review in question. Working independently, two coders will extract data from each included review, using the form shown in Supporting Information: Appendix 3. For each review, we will extract the following type of information:

- Descriptive information: publication year, update status, coordinating group(s), co‐registration status (with Cochrane)
- Included study characteristics: included study designs (RCT only, RCT and other), numbers of included studies
- ORB assessment methods (if any)
- Analysis and discussion of ORB

Bibliographic data will be stored in Zotero. Extracted data will be stored in excel spreadsheets. All coders will independently pilot test the data extraction form and revisions will be made as needed.

#### 3.3.3 Assessment of risk of bias

We will extract data on whether and how Campbell SRs assessed risks of ORB in the primary studies they included (see Supporting Information: Appendix 3). For each SR, we will record how many primary studies were included; whether any studies were excluded because review outcomes were not reported; how many were assessed as low risk of bias, high risk, or unclear risk; and how these judgments were defined and supported.

#### 3.3.4 Unit of analysis issues

The primary unit of analysis is the SR. Within SRs, we plan to collect and analyze data on reviewers’ coding and classification of included studies.

#### 3.3.5 Dealing with missing data

We will contact review authors in cases where there is missing or incomplete information.

#### 3.3.6 Data synthesis

We will use descriptive statistics (frequencies and percentages) to describe included reviews, the methods they used to assess ORB, and the results of these assessments. Aggregated data will be presented in tables and graphs.

As we are not synthesizing effect sizes, there will be no MA.

#### 3.3.7 Subgroup analysis and investigation of heterogeneity

If sufficient data are available, we will report differences between SRs that limited included studies to RCTs versus those that included other study designs (perhaps in addition to RCTs). RCTs are more likely to have pre‐registered protocols than other kinds of studies. Thus, the practice of ORB assessment may vary across SRs, depending on the types of studies they included.

If sufficient data are available, we could report results separately for each Coordinating Group (CG). CGs produce SRs in different substantive domains within the Campbell Collaboration (e.g., Crime and Justice, Education, Social Welfare, International Development). Therefore, their reviews may reflect research norms in different substantive fields.

With sufficient data, we could explore between‐group differences (e.g., proportion of reviews that did not assess ORB, proportion that used study protocols to assess ORB, proportion that provided justification for each ORB rating) using moderator analysis. We do not expect to have enough reviews in this study to support such analyses.

#### 3.3.8 Sensitivity analysis

We do not plan to conduct sensitivity analysis.

#### 3.3.9 Treatment of qualitative research

We will extract qualitative data (sentences and phrases) from included reviews to capture definitions of ORB verbatim. When possible, similar definitions will be collapsed into categories for descriptive analysis. We will use selected verbatim definitions to illustrate different approaches.

#### 3.3.10 Summary of findings and assessment of the certainty of the evidence

We will summarize findings in tables, graphs, and narratives. The structure of this summary will follow the questions posed in the section on Objectives. We do not plan to include an assessment of the certainty of the evidence.

Our review may lead to recommendations for improving future assessments of ORB in SRs. This might include guidance for review authors and editors, as well as suggested modifications of ROB assessment tools, and directions for further research on this topic.

## CONTRIBUTIONS OF AUTHORS

All members of the review team have expertise in the content area, systematic review methods, statistical analysis, and information retrieval.

JL and DG conceived the idea for this project. JL drafted the protocol; DG, JV, and TP reviewed and commented on the protocol.

## DECLARATIONS OF INTEREST

JL, TP, and JV are co‐authors of one or more published Campbell reviews. No member of the review team will be involved in screening or extracting data from any reviews they co‐authored.

## PRELIMINARY TIMEFRAME

The first draft of the review will be completed by December 2023.

## PLANS FOR UPDATING THIS REVIEW

The review will be updated once every 5 years, if sufficient resources are available. All members of the review team will participate in updates, depending on their availability.

## SOURCES OF SUPPORT

**Internal sources**

- None, Other

None.

**External sources**

- None, Other

None.

## Supporting information

Supporting information.
