# Supplementary material for: PROTOCOL: Assessment of outcome reporting bias in studies included in Campbell systematic reviews
Source: Campbell Syst Rev. 2023 May 25;19(2):e1332. doi: 10.1002/cl2.1332 (PMC10210598; doi:10.1002/cl2.1332)
Supplement: Supplementary file 1 — Supporting information. [file CL2-19-e1332-s001.pdf]

Appendix 1: Search strategy

Source: Campbell Library search form (available at: https://www.campbellcollaboration.org/component/jak2filter/?Itemid=1352&issearch=1&isc=1&category_id=101&xf_8[0]=3&ordering=publishUp)

Filters

#	Filter type	Filter contents
1	Published date	2020-01-01 to 2022-12-31
2	Type of document	Review (only)

Appendix 2: Screening potentially eligible studies

1. Is this a systematic review of *intervention effects*?

    1 = Yes
    0 = No [EXCLUDE]
    8 = Unclear

2. Does this review include *primary studies*?

    1 = Yes
    0 = No, it is a review of reviews [EXCLUDE]
    8 = Unclear

Appendix 3: Data extraction form

Basic information

1. Publication year: 2020, 2021, 2022

2. Update status: Is this an update of a previous Campbell review?
   1 = Yes
   0 = No

3. Campbell Coordinating Group (if co-registered, check all that apply):
   - Business & Management
   - Crime & Justice
   - Disability
   - Education
   - International Development
   - Knowledge Translation and Implementation
   - Methods
   - Nutrition
   - Social Welfare
   - Other, name: ___________

4. Was the review co-registered with Cochrane?
   1 = Yes (WHICH GROUP?),
   0 = No

Characteristics of included studies

5. Study design inclusion criteria
   1 = RCTs only
   2 = RCTs and other designs

6. Were any studies excluded because they did not report relevant outcomes?
   1 = Yes (EXPLAIN and document how many studies were excluded for this reason.)
   0 = No
   8 = Unclear

Number of studies in the completed review

Number of studies	RCTs	Non RCTs	Total
Met inclusion criteria			
Included in narrative synthesis			
Included in quantitative synthesis			

ORB assessment

7. Did the review include any structured, study-level assessment of risk of bias (ROB) or study quality (MECCIR C20, C51, R46)?
    1 = Yes
    0 = No
    8 = Unclear

8. Did the review include any structured, study-level assessment of ORB within included studies?
    1 = Yes
    0 = No [SKIP to Question 20]
    8 = Unclear

9. What criteria were used to assign studies to ORB risk categories (MECCIR R46)? That is, how were high risk, unclear risk, and low risk (or other categories) of ORB defined? [Extract quotations and page numbers.]

10. Did reviewers attempt to obtain study protocols (or other evidence of planned outcomes and analyses) from...

	Yes	No	Unclear
Public registries?			
Study authors?			
Other sources? [EXPLAIN]			

11. Did reviewers use study protocols (or other evidence of planned outcomes) as a source of data on ORB?
    1 = Yes
    0 = No
    8 = Unclear

12. Did reviewers attempt to distinguish prospectively registered protocols (or outcome lists) from retrospectively registered or revised (altered) protocols?
    1 = Yes [EXPLAIN: extract information on how these distinctions were made, record quotations and page numbers]
    0 = No
    8 = Unclear

13. How did reviewers assess ORB?
    a. Compared prospectively registered protocol/outcome list to reported outcomes? Y/N/Unclear
    b. Compared any protocol (prospective/retrospective) to reported outcomes? Y/N/Unclear
    c. Compared early report(s) to later reported outcomes? Y/N/Unclear
    d. Compared methods sections to outcomes reported in same document? Y/N/Unclear

e. Considered any reporting of “relevant” outcomes to be evidence of low risk of ORB? Y/N/Unclear
f. Other (explain)

14. Did reviewers assess risk of ORB in duplicate (MECCIR C52)?
2 = Yes for all
1 = Yes for some [HOW MANY STUDIES?]
0 = No
8 = Unclear

15. Did reviewers report inter-rater reliability of risk of ORB assessments?
1 = Yes [PROVIDE DETAILS: NUMBER OF STUDIES ASSESSED, KAPPA/ OTHER STATISTICS]
0 = No
8 = Unclear

16. Details of analysis

Number of studies assessed as	RCTs	Non RCTs	Total
High risk of ORB			
Unclear risk of ORB			
Low risk of ORB			
Other category/ies (DESCRIBE)			
Not assessed (missing)			

17. Did reviewers provide study-level documentation (evidence or explanations) for their ORB ratings (MECCIR C53, R72)?
2 = Yes for all
1 = Yes for some (HOW MANY?)
0 = No
8 = Unclear

18. Did reviewers identify sources of support for ORB judgements (MECCIR C54)? (e.g., quotations and page numbers from study documents)
2 = Yes for all
1 = Yes for some [HOW MANY?]
0 = No [SKIP TO...]
8 = Unclear

19. If yes (for all or for some), what type of support was provided for ORB assessments? Check all that apply:
a. quotes from source material? Y/N
b. page numbers from source material? Y/N
c. other? (explain) Y/N

Analysis and discussion of ORB

20. Did reviewers provide a narrative summary of risks of ORB (MECCIR R74)? (This is usually in the Results section.)
    1 = Yes
    0 = No
    8 = Unclear

21. Was there any moderator analysis using risk of ORB?
    1 = Yes (DESCRIBE analysis and results)
    0 = No
    8 = Unclear

22. Did reviewers discuss potential impacts of ORB on review findings (MECCIR R89, R95, R96)?
    1 = Yes (record page numbers)
    0 = No
    8 = Unclear

23. Were issues of ORB mentioned (at all) in the:

	ORB mentioned?
Abstract	Y/N/Unclear
Plain Language Summary	Y/N/Unclear
Limitations	Y/N/Unclear
Conclusions	Y/N/Unclear
Other (describe)	Y/N/Unclear

24. Were issues related to other ROB (not ORB) mentioned (at all) in:

	Other risks of bias mentioned?
Abstract	Y/N/Unclear
Plain Language Summary	Y/N/Unclear
Limitations	Y/N/Unclear
Conclusions	Y/N/Unclear
Other (describe)	Y/N/Unclear
